# No Evidence Supports the Presence of SRF-type MADS-Box Genes in Land Plants

**DOI:** 10.1093/gbe/evag140

**Published:** 2026-06-16

**Authors:** Yichun Qiu, Zhen Li, Claudia Köhler

**Affiliations:** Department of Plant Reproductive Biology and Epigenetics, Max Planck Institute of Molecular Plant Physiology, Potsdam 14476, Germany; Department of Plant Biotechnology and Bioinformatics, Ghent University, Ghent, Belgium; VIB Center for Plant Systems Biology, VIB, Ghent, Belgium; Department of Plant Reproductive Biology and Epigenetics, Max Planck Institute of Molecular Plant Physiology, Potsdam 14476, Germany; Department of Plant Biology, Uppsala BioCenter, Swedish University of Agricultural Sciences and Linnean Centre for Plant Biology, Uppsala 75007, Sweden

**Keywords:** MADS-box transcription factors, Type I, MEF2-type, SRF-type, land plant

## Abstract

MADS-box transcription factors are a key gene family central to understanding the evolutionary success of land plants. Land plant MADS-box genes are divided into Type I and Type II. They were initially considered orthologous to SRF and MEF2 genes in animals and fungi, respectively, which originated by an ancient duplication before the diversification of extant eukaryotes. However, using updated phylogenetic analyses and AI-based protein structure prediction, we showed that both Type I and Type II genes are plant-specific duplicates derived from MEF2-type ancestors, while ancestral SRF genes were lost before the divergence of the plant lineage. A recent study proposed instead a polyphyletic origin for plant Type I genes. While this study also found that the majority of Type I MADS-box genes in land plants cluster with MEF2 genes, a few genes were grouped as Type I and considered related to SRF genes. By reanalyzing the original data from that study, we do not find supporting evidence for this hypothesis. Moreover, using phylogeny, sequence similarity, and structural evidence, we demonstrate that there is no evidence of SRF-derived genes in land plants.

Significance Two major clades of MADS-box transcription factors, SRF and MEF2, originated from an ancient duplication event and are widely distributed across extant eukaryotes. We previously demonstrated that all MADS-box genes in land plants belong to the MEF2 clade and inferred that SRF-type genes were absent from the most recent common ancestor of the plant lineage. In contrast, another study recently proposed that a small subset of land plant MADS-box genes is derived from SRF-type ancestors. Here, we present a comprehensive reanalysis of the data reported in that study, together with additional phylogenetic, comparative sequence, and structural evidence, and find no support for the claim of SRF-origin. Our results reinforce the conclusion that SRF-type MADS-box genes are not present in land plants and clarify the evolutionary history of this key transcription factor family. This work further advocates the requirement of caution when interpreting gene family phylogenies encompassing diverse sequences.

MADS-box genes encode transcription factors (TFs) that have long fascinated the plant science community, due to their crucial roles in regulating plant development, especially reproductive processes in flowering plants (Kaufmann et al. 2005; Theißen et al. 2016; Qiu and Köhler 2022). This gene family underwent substantial expansion across land plant lineages (Nam et al. 2003; Gramzow and Theißen 2013; Qiu et al. 2023). Based on the arrangement of conserved domains, MADS-box genes are traditionally categorized into M-type and MIKC-type, the latter characterized by a plant-specific Keratin-like (K) domain (Alvarez-Buylla et al. 2000; Parenicová et al. 2003). Many plant MIKC-type TFs have been initially identified as regulators of floral organ identity and flowering time (Kaufmann et al. 2005; Theißen et al. 2016). They were later specified as the “classic” MIKC^C^-type, after a new group of MIKC*-type TFs was characterized, which can be distinguished further by the K domain structure (Henschel et al. 2002). MIKC*-type TFs majorly function during the development of the male gametophyte, the pollen (Verelst et al. 2007). M-type TFs lack a K domain and have been grouped into Mα, Mβ, and Mγ subclades based on phylogenetic clustering (Parenicová et al. 2003). Many Mα and Mγ TFs are key regulators of female gametophyte and endosperm development (Qiu and Köhler 2022). Flowers, fruits, pollen, and endosperm are major reproductive innovations that contributed to the evolutionary success and diversification of flowering plants. Consequently, understanding the evolutionary history of these MADS-box genes has long been of considerable interest and importance.

Following the identification of M-type genes in the Arabidopsis genome, a pioneering study investigated the evolutionary history of eukaryotic MADS-box genes and proposed that plant M-type genes, represented by those in Arabidopsis, are putatively related to animal SRF and yeast MCM1 genes, while plant MIKC-type genes are orthologous to animal MEF2 genes (Fig. 1a; Alvarez-Buylla et al. 2000). These two groups became known as Type I and Type II, a classification widely adopted in the plant science community and long maintained. Like many other TFs, MADS-box TFs contain relatively few informative characters for robust phylogenetic inference. Therefore, it is often challenging to reach well-resolved phylogenies across vast evolutionary distances, e.g. throughout eukaryotes, a difficulty that was even more pronounced in earlier studies due to sparse sequence data. Under similar data limitations, several previous studies also reported conflicting phylogenies, particularly regarding the origin and evolution of M-type genes, which historically received less attention (de Bodt et al. 2003, Kofuji et al. 2003).

**Fig. 1.**
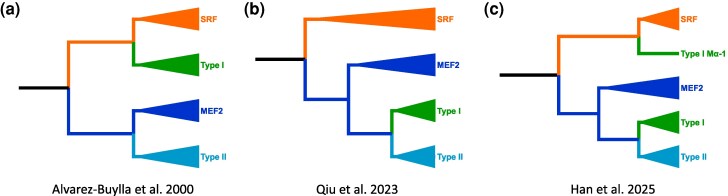
Three models of MADS-box transcription factor (TF) gene evolution. a) The original model by Alvarez-Buylla et al. (2000) proposed that plant Type I and Type II TFs are orthologous to animal SRF TFs and MEF2 TFs, respectively. b) Qiu et al. (2023) revised the previous hypothesis: SRF TFs are not present in plants, and both plant Type I and Type II TFs are plant-specific duplicated clades derived from MEF2 TFs. c) Han et al. (2025) proposed a polyphyletic origin of plant Type I TFs by suggesting that a subset of Type I TFs is derived from SRF genes. However, we identified multiple lines of evidence showing that this recently proposed model is not supported.

The rapid expansion of genomic and transcriptomic data across all major eukaryotic linages has provided an excellent opportunity to refine these earlier investigations, particularly through the inclusion of sequences from charophytes, the closest algal relatives of land plants (Kunz et al. 2025). Recently, we challenged the prevalent hypothesis of MADS-box gene evolution by an updated phylogenetic analysis of MADS-box genes from 175 sequenced genomes, assisted by their protein structures (Qiu et al. 2023). Our results show that SRF-type genes were lost in the plant lineage, and that both M-type and MIKC-type genes, corresponding to Type I and Type II, respectively, are streptophyte-specific duplicates of MEF2-type genes, likely arising after the divergence from chlorophytes (Fig. 1b; Qiu et al. 2023). Building on the discovery of an MEF2-only origin for plant MADS-box genes, we further reexamined the relationships among subtypes of MIKC-type genes (Qiu et al. 2024). We reclassified MIKC*-type genes within the updated Type I clade, based on their closer clustering with M-type genes, while the MIKC^C^-type genes constitute a refined Type II clade, which diverged following an ancestral MEF2 gene duplication giving rise to the Type I and II sister clades (Qiu et al. 2024). The new phylogeny nicely reflects the functional divergence of MADS-box genes in flowering plants, with Type I TFs largely retaining ancestral roles in gametophyte development and acquiring additional function in the endosperm, while Type II TFs evolved novel functions associated with sporophyte development.

Han et al. (2025) reassessed our proposed model of MADS-box gene evolution by expanding the dataset to include MADS-box sequences from 551 eukaryotic species, covering 294 whole genomes and 257 transcriptomes. Using this comprehensive data, Han et al. identified over 1000 M-type genes and categorized most of them into three previously established subgroups (Parenicová et al. 2003), 558 Mα, 201 Mβ, and 257 Mγ genes in land plants. The majority, 510 Mα (91.4%), all Mβ, and all Mγ genes were identified as MEF2-type, thus providing strong support for our conclusion in Qiu et al. (2023). However, Han et al. proposed a polyphyletic origin of M-type MADS-box genes by classifying 48 Mα genes as SRF-type, designated “Mα-1”, in contrast to the majority of MEF2-type Mα genes, referred to as Mα-2 (Fig. 1c).

We find these claims contain internal inconsistencies that call their logic into question. It remains unclear how the 48 “Mα-1” genes were classified as Mα-type based on homologous alignments and structural analyses, as Han et al. (2025) provided only a vague explanation. If these “Mα-1” genes were truly SRF-type, they would be distantly related to Mα-2 genes, which cluster with Mβ and Mγ genes instead. Therefore, assigning them as a subtype of Mα group is inconsistent and unjustified, since the three clades, Mα, Mβ and Mγ, were initially defined by their close phylogenetic relationship (Parenicová et al. 2003). Moreover, the sporadic distribution of “Mα-1” genes across discrete plant lineages challenges their SRF origin. Of the 48 genes, the 11 angiosperm sequences are restricted to just four (*Oryza sativa*, *Brachypodium distachyon*, *Prunus persica,* and *Vitis vinifera*) of 19 surveyed species. Such a pattern would imply repeated, independent losses of “Mα-1” genes in the other 15 angiosperms, a theoretically possible but evolutionarily less parsimonious scenario. Additionally, among gymnosperms (19 sequences), ferns (nine sequences), and lycophytes (nine sequences), “Mα-1” genes were identified only in a minority of surveyed species. Notably, none are detected in 54 bryophyte genomes. This distribution pattern of “Mα-1” genes contrasts sharply with what would be expected for a conserved SRF lineage. While the most parsimonious model might not reflect the true evolutionary pattern, careful evaluation and high-quality evidence should be considered for a hypothesis in conflict with the principle of parsimony. Therefore, we found it important to reevaluate the data in Han et al. (2025). Here, we present multiple lines of evidence against this interpretation of SRF-derived “Mα-1” genes in land plants and argue that the proposed polyphyletic origin of plant M-type genes remains unsupported.

First, Han et al. misinterpreted the unrooted maximum likelihood trees (Fig. 1c and d of Han et al. 2025), in which the nonplant SRF genes seemed to group together with “Mα-1” sequences from land plants into a single clade. These classifications are arbitrary and confounded by misleading tree visualizations.

For the three trees with outgroups (shown in Fig. 1d of Han et al. 2025), by re-rooting them using the outgroups, all the nonplant SRF genes form a well-supported clade, but the MEF2-derived genes are no longer monophyletic, in contrast to the current understanding and the claim by Han et al. In these cases, MEF2-derived genes and “Mα-1” genes become successive sister groups of the nonplant SRF genes, so one cannot simply group “Mα-1” genes into SRF-type while arbitrarily ignoring the neighboring MEF2-derived genes (Fig. 2a; Figs. S1 and S2). The misinterpretation issue is further compounded by the choice of inappropriate outgroups. Han et al. used bacterial topoisomerase IIA subunit A (TOPOIIA) proteins, bacterial universal stress proteins (USPs), plant APETALA2/ethylene-responsive element binding proteins (AP2/EREBPs), and plant basic region/leucine zipper motif (bZip) proteins as outgroups. However, only a subset of TOPOIIAs were reported to be putatively homologous to MADS-box genes (Gramzow et al. 2010), and USPs have been suggested to be suboptimal for such analyses (Alvarez-Buylla et al. 2000). Moreover, AP2 and bZIP sequences are not homologous to MADS-box genes. The lack of homologous sites in the outgroup sequences may have misled the multiple sequence alignments of the ingroup sequences, leaving untrustworthy phylogenetic relationships for the ingroup. As a result, these outgroups consistently form long-branched clades arising from inconsistent positions across phylogenies, and in two cases, they formed more than one monophyletic group, making it difficult to re-root the inferred phylogeny.

**Fig. 2.**
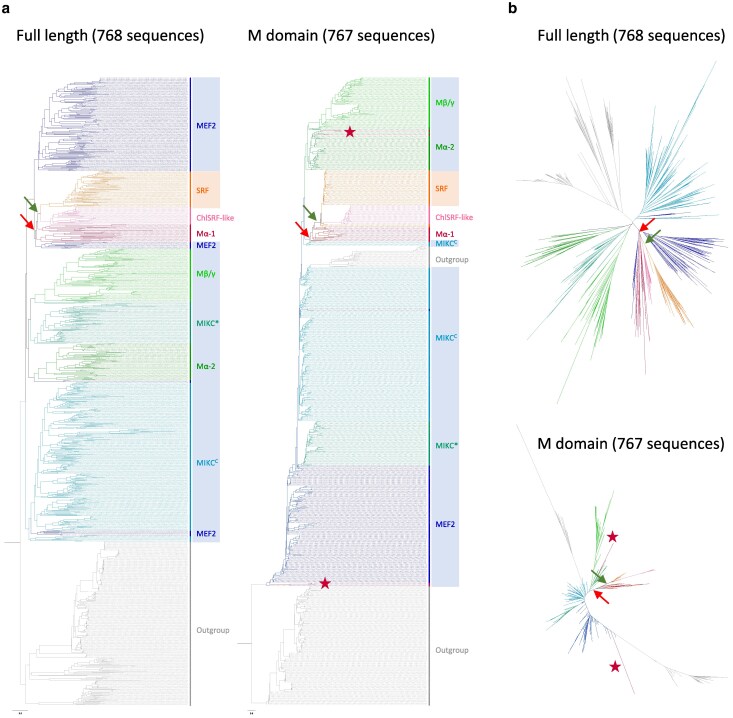
The redrawn trees of the MADS-box protein phylogeny shown in Fig. 1d of Han et al. (2025). a) The trees rooted by the outgroups. While all SRF sequences (orange background) form a clade (marked by the green arrow), “Mα-1” sequences together with confirmed MEF2-derived MADS-box sequences (blue background) are paraphyletic, and become successive sisters to the SRF clade. Han et al. (2025) arbitrarily grouped the “Mα-1” sequences into an unjustified clade along with SRF sequences (marked by the red arrow). The ChlSRF-like clade comprises SRF-like sequences in chlorophytes. Sequence IDs and taxon color codes are the same as the expanded tree in Fig. S2. b) The unrooted trees. “Mα-1” sequences position between the SRF clade and the MEF2 clade. Red stars mark the “Mα-1” sequences nested in the MEF2-type clade.

Alternatively, to treat them as unrooted trees by ignoring the problematic outgroups, together with the unrooted tree without outgroups (shown in Fig. 1c of Han et al. 2025), we found that nearly all of the nonplant SRF genes form a well-supported clade by themselves, while the land plant “Mα-1” genes did not nest within the SRF clade, nor did they form a single monophyletic group. Instead, most of the “Mα-1” genes are dispersed as small and long-branched clades, between the SRF clade and the MEF2 clade, the latter of which includes other land plant MADS-box genes (Fig. 2b; Figs. S1–S3). This positioning does not allow us to conclude whether these “Mα-1” genes are related to SRF or MEF2. Furthermore, some “Mα-1” genes (e.g. two from *Oryza sativa*, two from *Vitis vinifera*, and four from *Selaginella moellendorffii*) cluster within MEF2 clades in multiple trees, directly contradicting the claim of their origins within the SRF clade, which was surprisingly neglected in the original interpretation (Figs. S1 and S2). In all four trees, “Mα-1” genes and a couple of their close sister sequences are long branches (Fig. 2b; Figs. S1–S3). Such long branches may reflect high divergence, but they are also a well-known source of phylogenetic artifacts and ambiguities, where unrelated sequences tend to cluster together, as specified as long branch attraction (Felsenstein 1978). Similarly, SRF-like genes in core chlorophytes also remain questionable, which are only loosely similar to SRF sequences as previously noted (Nayar and Thangavel 2021; Qiu et al. 2023).

Since Han et al. did not present a phylogeny including all 48 “Mα-1” genes, we used their datasets to reconstruct such a maximum likelihood tree using IQ-TREE2 (Minh et al. 2020), following the same methods as Qiu et al. (2023). Our result shows that 11 angiosperm “Mα-1” genes cluster within the MEF2 clade alongside other M-type genes, while the remaining 37 nonangiosperm sequences remain ambiguous due to long branches located between the clearly defined MEF2 and SRF clades (Fig. 3; Fig. S4). This finding suggests that some “Mα-1” genes are best considered divergent MEF2-type sequences, rather than SRF-derived, and some are too divergent in sequences to infer the most closely related homologs. Together, the seemingly relatedness of SRF genes and the “Mα-1” sequences cannot be taken at face value.

**Fig. 3.**
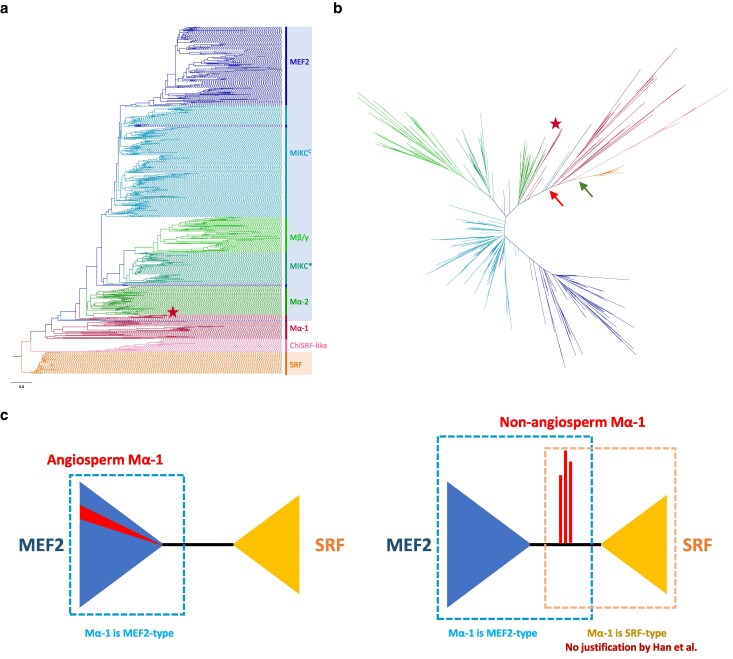
The maximum likelihood tree of MADS-box proteins in eukaryotes. All the 48 “Mα-1” sequences are included in addition to other MADS-box sequences as in Table S5 from Han et al. (2025). The alignment is available in Supplementary Note S1. a) The tree rooted by the SRF clade for visualization purposes. Confirmed MEF2-derived sequences have a blue background, and SRF sequences have an orange background. The ChlSRF-like clade comprises SRF-like sequences in chlorophytes. Sequence IDs and taxon color codes are the same as the expanded tree in Fig. S4. b) The unrooted tree. On the right side of the green arrow are the SRF sequences, and on the left side of the red arrow are the MEF2-derived sequences. Red stars mark the “Mα-1” sequences nested in the MEF2-type clade. c) Illustrations of the 11 angiosperm “Mα-1” proteins and the 37 nonangiosperm “Mα-1” proteins in the phylogeny.

Furthermore, Han et al. used BLASTP to compare sequence similarities between “Mα-1”, SRF, and MEF2 proteins. We argue that sequence similarities cannot reliably determine evolutionary origins, especially for distantly related or divergent sequences. Even within this suboptimal framework, the analyses by Han et al. (2025) remain questionable. Aside from the lack of a clear description of the analyzed sequences and justification for the sampling strategy, the massive number of pairwise comparisons further obscures informative signals. Not all BLASTP hits are biologically meaningful, especially those with large E-values, some of which are even larger than 1. We also performed BLASTP analyses using the 48 “Mα-1” proteins from Han et al. (2025) as queries, against other types of MADS-box sequences used in the sequence logos in Han et al. (2025), ensuring the same size and composition of the subject database (Table S1). Using more biologically relevant indicators, such as top BLAST hits, we found that the 11 angiosperm “Mα-1” proteins consistently match MEF2-derived M-type proteins, aligning with our phylogenetic results above. For the 37 nonangiosperm “Mα-1” proteins, top hits were more ambiguous and often had high *E*-values, making it not possible to assign closely related sequences. Nonetheless, these top hits for a given nonangiosperm “Mα-1” protein were either dominated by MEF2-type sequences or represented mixed SRF and MEF2 matches with negligible differences in *E*-values. Therefore, the BLASTP analyses from Han et al. (2025) provide no convincing evidence for an SRF origin of the 48 “Mα-1” genes. Instead, BLASTP results only reflect that these “Mα-1” sequences are more divergent from other MADS-box proteins (Fig. S5).

Last, our previous protein structural analyses provided further evidence for a sole MEF2 origin of M-type and MIKC-type genes (Qiu et al. 2023). Functional MADS-box transcription factors bind DNA as dimers, requiring two α-helices connected by two antiparallel β-strands (Fig. 4; Fig. S5). The canonical MADS domain forms the first helix and β-strands, while the second helix is provided by the SAM domain (in SRF), the MEF2 domain (in MEF2), or the Intervening (I) domain (in plants). We extended the definition of the MADS domain to include this second helix, which is essential for dimerization in all functionally characterized MADS-box TFs (Lai et al. 2021; Qiu et al. 2023). Resolved protein structures reveal distinct orientations of this helix in SRF versus MEF2 proteins, which can be nicely captured in ΑlphaFold-predicted structures as we previously demonstrated (Qiu et al. 2023). Applying this approach, we predicted the structures of the 48 “Mα-1” proteins. The 11 angiosperm “Mα-1” proteins consistently resemble the MEF2 structure rather than SRF, again challenging an SRF origin (Fig. 4; Figs. S5 and S6). For the 37 nonangiosperm “Mα-1” proteins, the second helix was absent or poorly predicted, preventing confident assignment to either SRF or MEF2 (Fig. 4; Figs. S5 and S6). This absence may indicate that, although the canonical MADS domain can be detected, these sequences have lost functionality as conserved MADS-box transcription factors due to the loss of protein–protein interacting interfaces. Just like the BLASTP results, these protein structure predictions alone are not deciding evidence for the origins. We consider that the variation in protein structures, consistent with the highly divergent sequences in the canonical MADS domain (Fig. S5), may explain why it is difficult to resolve the phylogenetic positioning of these “Mα-1” genes. Based on these observations, we hypothesize that these divergent “Mα-1” genes may have undergone neofunctionalization or pseudogenization, which would be interesting to test in future studies.

**Fig. 4.**
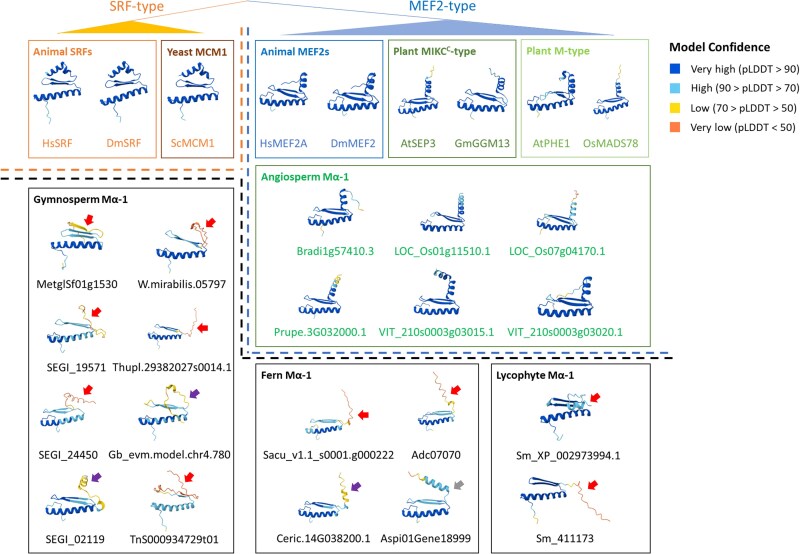
AlphaFold2-predicted structural models of selected MADS domains. The functional unit of MADS-box transcription factors forms two alpha helices and the connecting beta strands, where the second helix in SRF is twisted by a unique kink and oriented opposite to that in MEF2. Angiosperm “Mα-1” proteins resemble the MEF2 structure. For the other “Mα-1” proteins, red arrows mark the absence of a conserved helix, purple arrows mark the predicted helices with low confidence, and grey arrows mark the helix without a kink.

In summary, our reassessment of Han et al. (2025) indicates that there is no robust evidence for the presence of SRF-type MADS-box genes in land plants, nor for a polyphyletic origin of Type I genes. Among the 48 “Mα-1” genes ascribed to SRF by Han et al. (2025), the 11 angiosperm “Mα-1” genes are relatively divergent but functional MEF2-type M-type genes, as predicted by our earlier findings (Qiu et al. 2023). The remaining 37 nonangiosperm sequences are too divergent to be classified confidently, but can represent degenerated MEF2-derived sequences. Based on our previous findings, the absence of SRF genes in land plants remains the current null hypothesis (Qiu et al. 2023). This hypothesis cannot be conclusively proven, as absence itself is inherently difficult to demonstrate; rather, one can only demonstrate the failure to detect evidence for presence. It remains possible that this null hypothesis could eventually be rejected if robust evidence supporting an alternative interpretation emerges. However, the analyses presented by Han et al. are insufficient to reject this null hypothesis, given the aforementioned limitations in the dataset and methodology. Finally, should *bona fide* SRF-derived MADS-box genes ever be identified in land plants, they would represent a distinct, previously unrecognized lineage and should preferably be given a distinguishable designation from the well-characterized MEF2-derived M-type genes.

## Supplementary Material

evag140_Supplementary_Data

## Data Availability

The data underlying this article are available in the article and in its online supplementary materials.
