# Supplementary material for: No Evidence Supports the Presence of SRF-type MADS-Box Genes in Land Plants
Source: Genome Biol Evol. 2026 Jun 16;18(6):evag140. doi: 10.1093/gbe/evag140 (PMC13281830; doi:10.1093/gbe/evag140)

Fig. S1 The redrawn trees of the MADS-box protein phylogeny shown in Fig. 1D of Han et al. (2025). A. The tree rooted by the largest outgroup clade. All SRF sequences (orange background) form a clade (marked by the green arrow). Notice that the seeming separation of MEF2-derived sequences (blue background) from the SRF and "Mα-1" sequences by one outgroup clade may lead to misinterpretation. Since the two small clades of suboptimal outgroups raise as long branches, their phylogeny positions cannot be trustable and these branches should be ignored. Still, "Mα-1" sequences together with confirmed MEF2-derived MADS-box sequences are successive sisters to the SRF clade. Han et al. (2025) arbitrarily grouped the "Mα-1" sequences with SRF sequences into a single clade (marked by the red arrow) defined altogether as SRF-type, though the basis for this classification was not clearly justified. ChlSRF-like clade comprises SRF-like sequences in chlorophytes. Sequence IDs and taxon color codes are the same as the expanded tree in Supplementary Fig. S2. **B. The unrooted tree.** "Mα-1" sequences position between the SRF clade and the MEF2 clade. Red stars mark the "Mα-1" sequences nested in the MEF2-type clade.

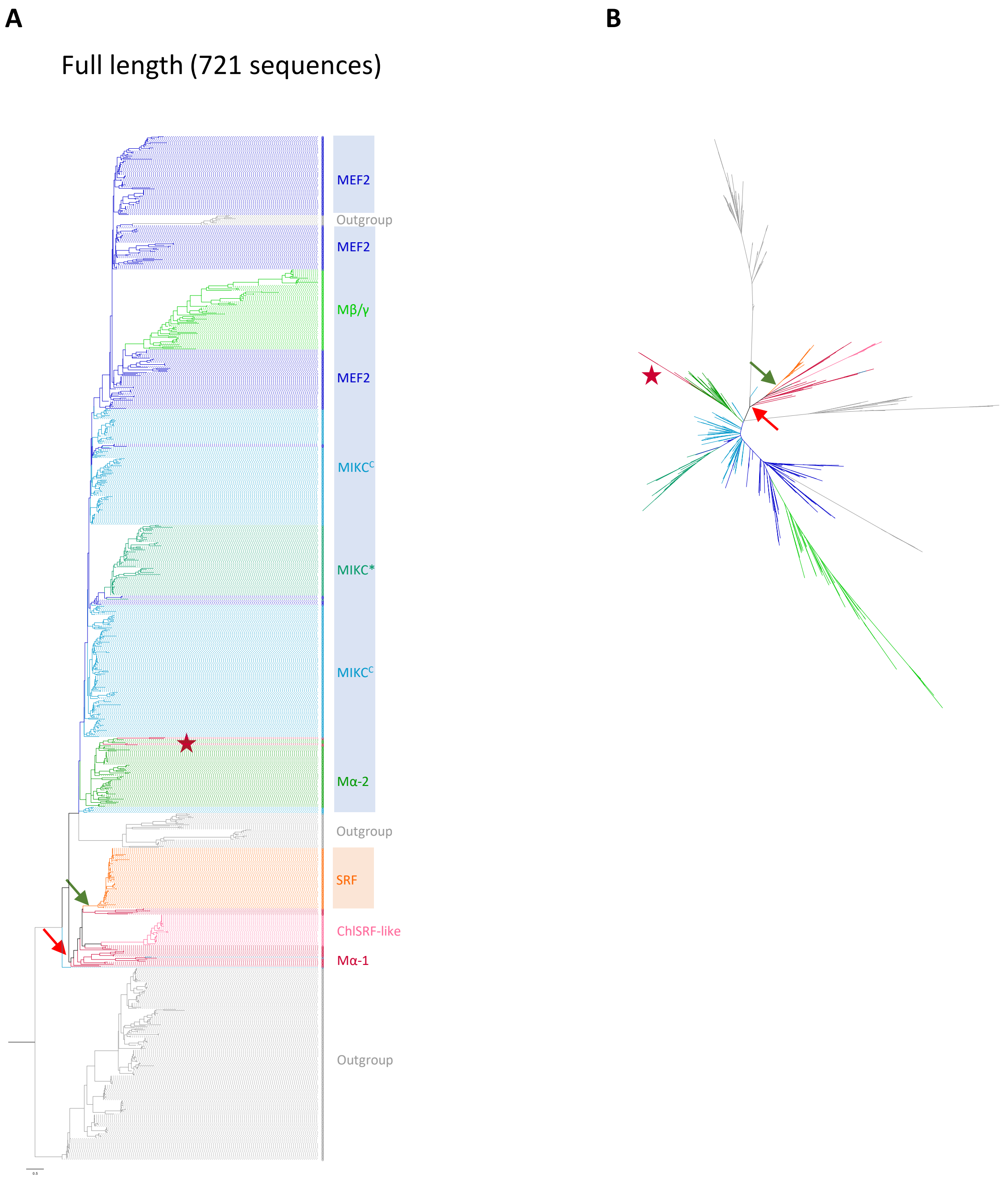

Fig. S2 The redrawn trees of the MADS-box protein phylogeny shown in Fig. 1D of Han et al. (2025). Rooted by the outgroups. Expanded to show gene IDs and branch support values for the trees in Fig.1 and Supplementary Fig. S1.

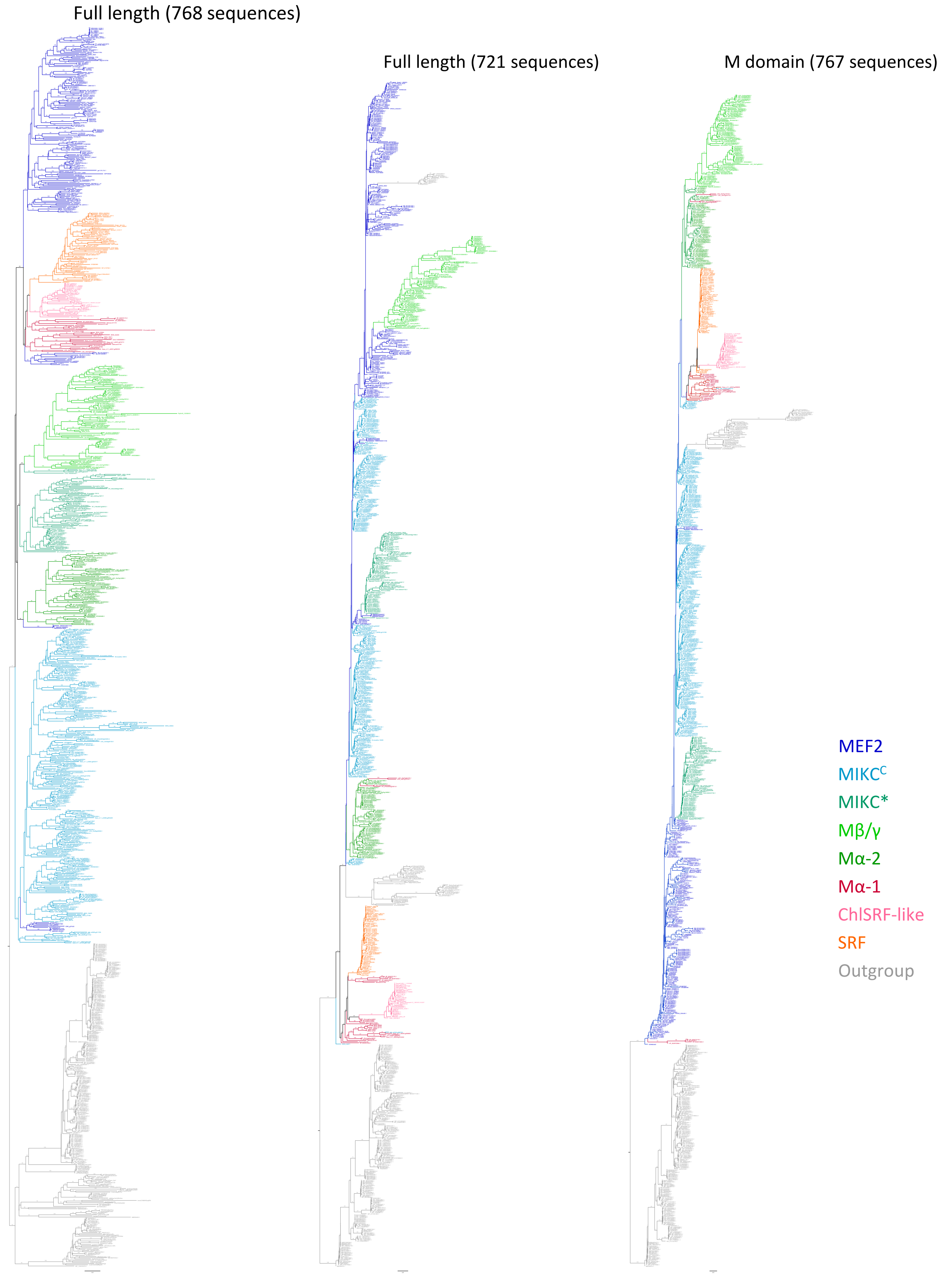

Fig. S3 The redrawn trees of the MADS-box protein phylogeny shown in Fig. 1C of Han et al. (2025). A. The unrooted tree. B. The tree rooted by the non-plant MEF2 clade. While nearly all SRF sequences form a clade (marked by the green arrow), “Mα-1” sequences together with confirmed MEF2-derived MADS-box sequences are successive sisters to the SRF clade. Han et al. (2025) arbitrarily grouped the “Mα-1” sequences into an unjustified clade along with SRF sequences (marked by the red arrow). “Mα-1” sequences position between the SRF clade and the MEF2 clade. Blue stars mark the clade of genes categorized as SRF-type in Han et al. (2025), which however, are identified as MEF2-type in Qiu et al. (2023).

B

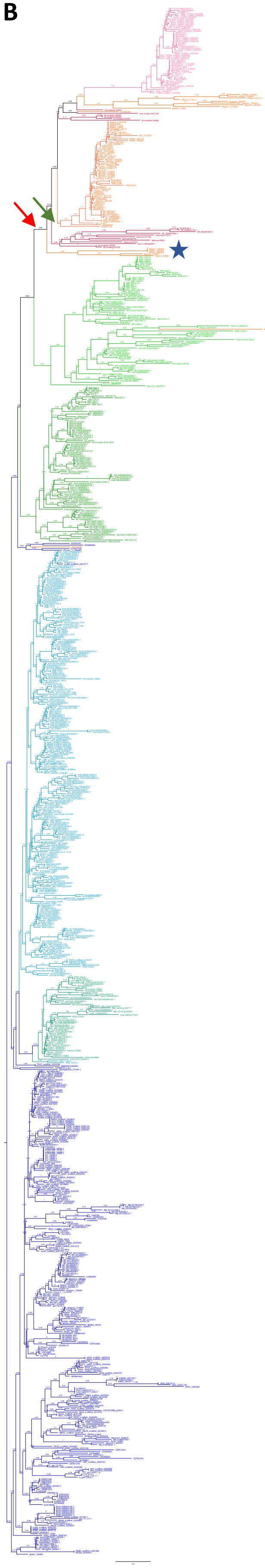

A

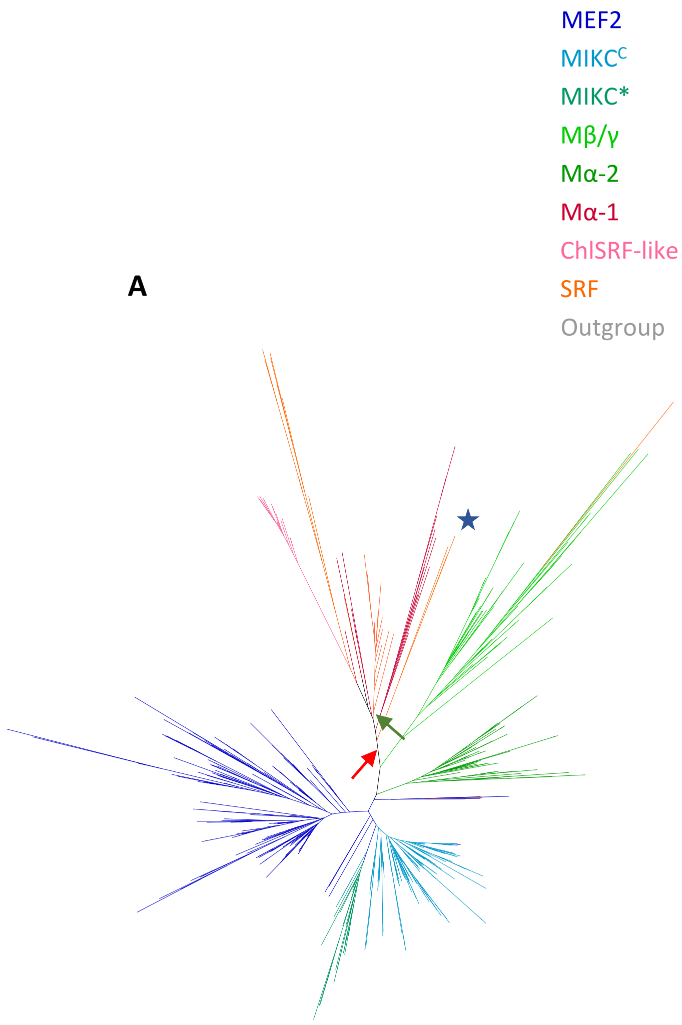

Fig. S4 The expanded tree of the MADS-box protein phylogeny shown in Fig.3. Rooted by the SRF clade. Gene IDs and branch support values are displayed correspondingly.

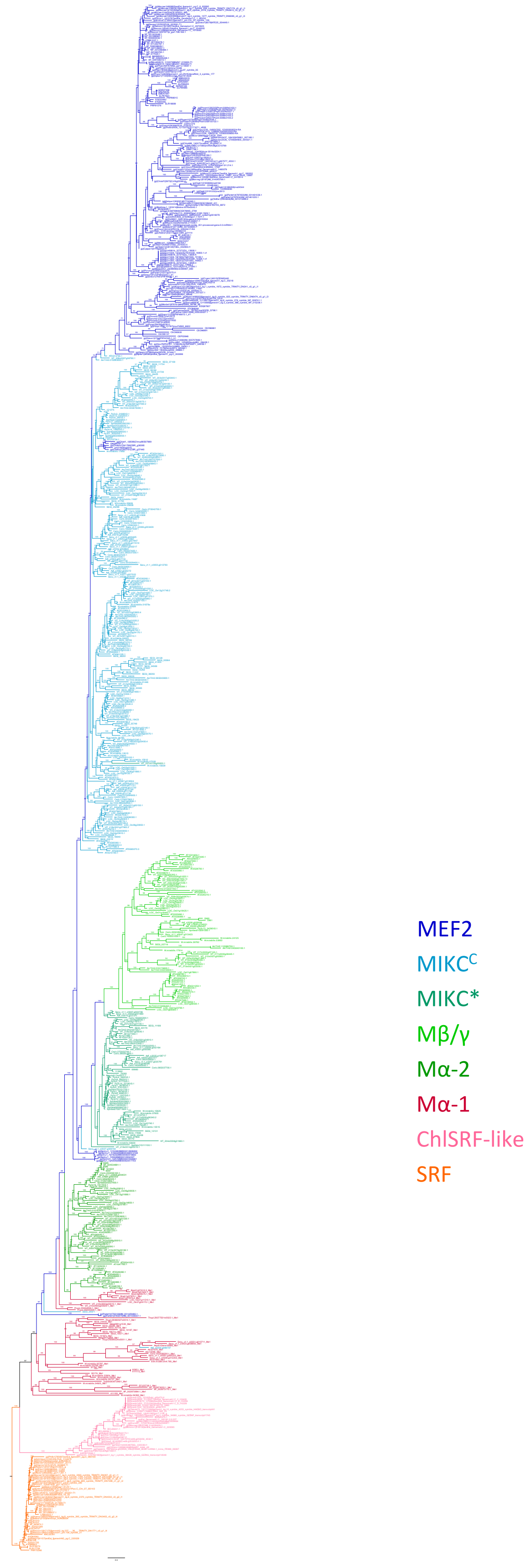

Fig. S5 The aligned MADS domain sequences from selected MADS-box transcription factors. 15 SRF and 15 MEF2 sequences are from animals, yeasts, and amoebae; 10 MIKC^{C}-type, 10 MIKC*-type, 10 Mβ/γ-type and 10 Mα-2 sequences are from all major lineages of land plants. "Mα-1" sequences are more divergent between each other and to the other types of MADS-box transcription factors. Gene IDs and sequences are listed in Supplementary Table 2.

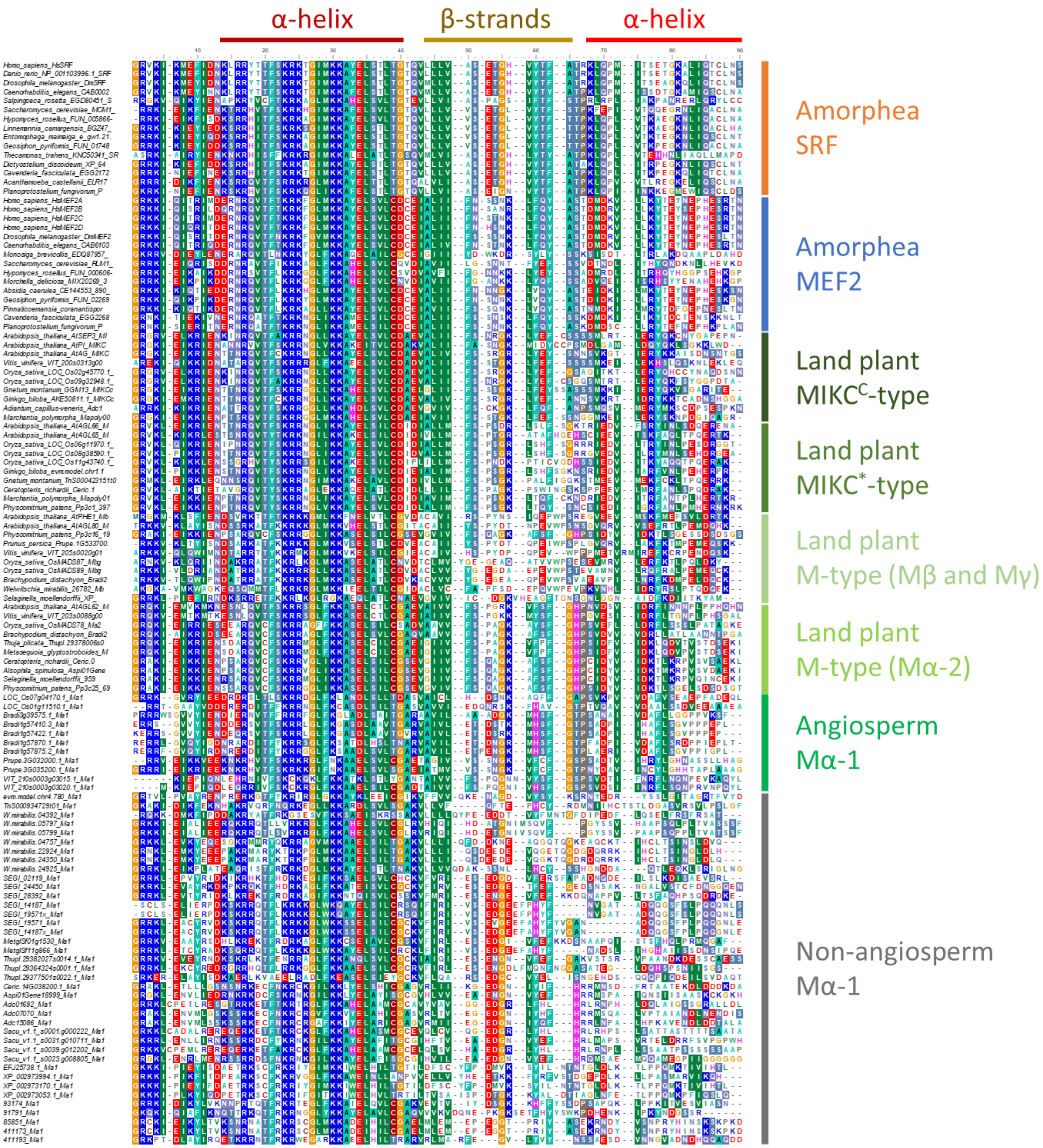

Fig. S6A AlphaFold2-predicted structural models of all 48 “Mα-1” MADS-box proteins (full-length). Red arrows mark the region after the beta strands where a helix is expected. Angiosperm “Mα-1” proteins resemble the MEF2 structure. For the other “Mα-1” proteins, the functional helix is either predicted absent or with low confidence.

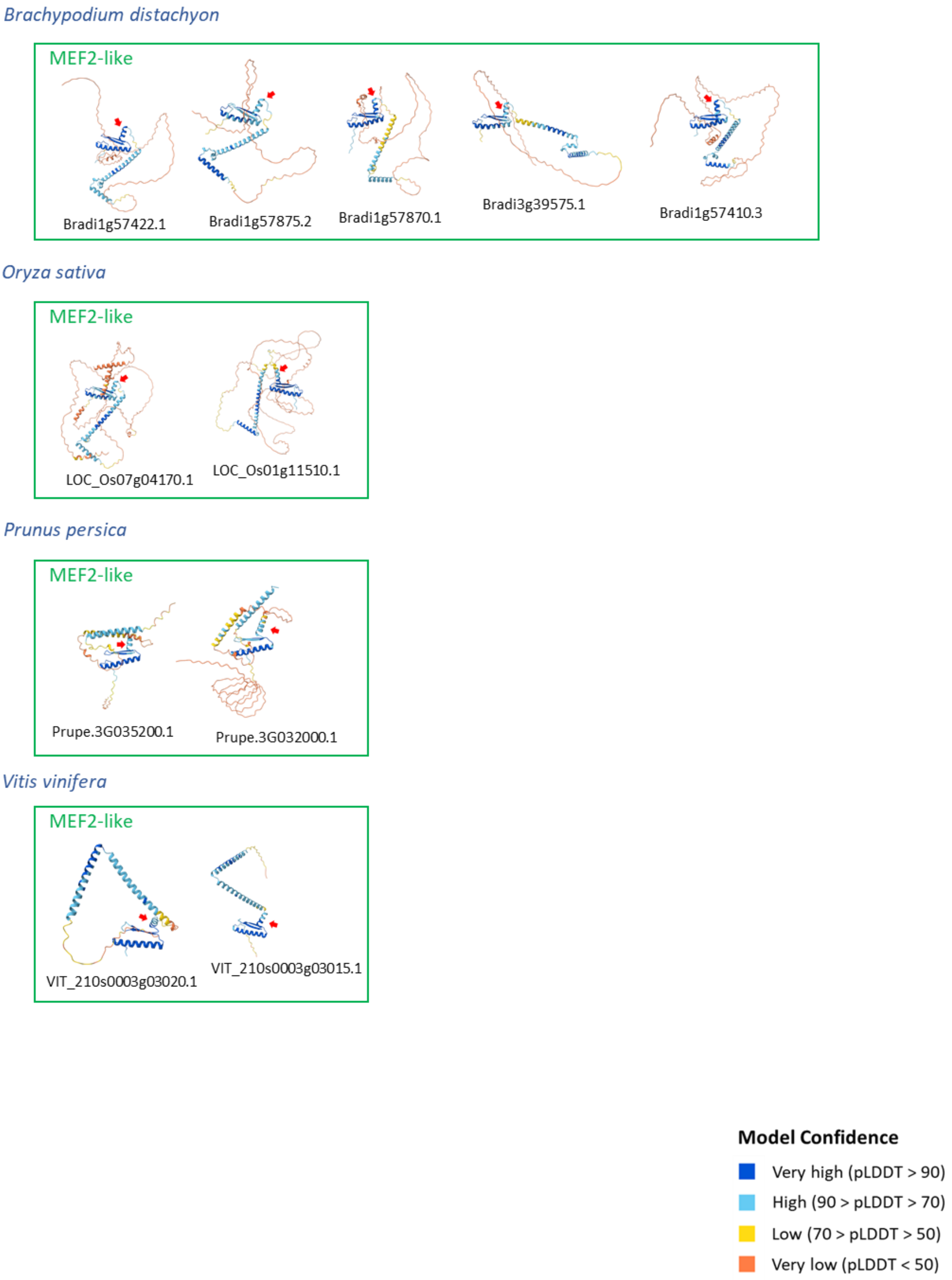

Fig. S6B AlphaFold2-predicted structural models of all 48 "Mα-1" MADS-box proteins (full-length). Red arrows mark the region after the beta strands where a helix is expected. Angiosperm "Mα-1" proteins resemble the MEF2 structure. For the other "Mα-1" proteins, the functional helix is either predicted absent or with low confidence.

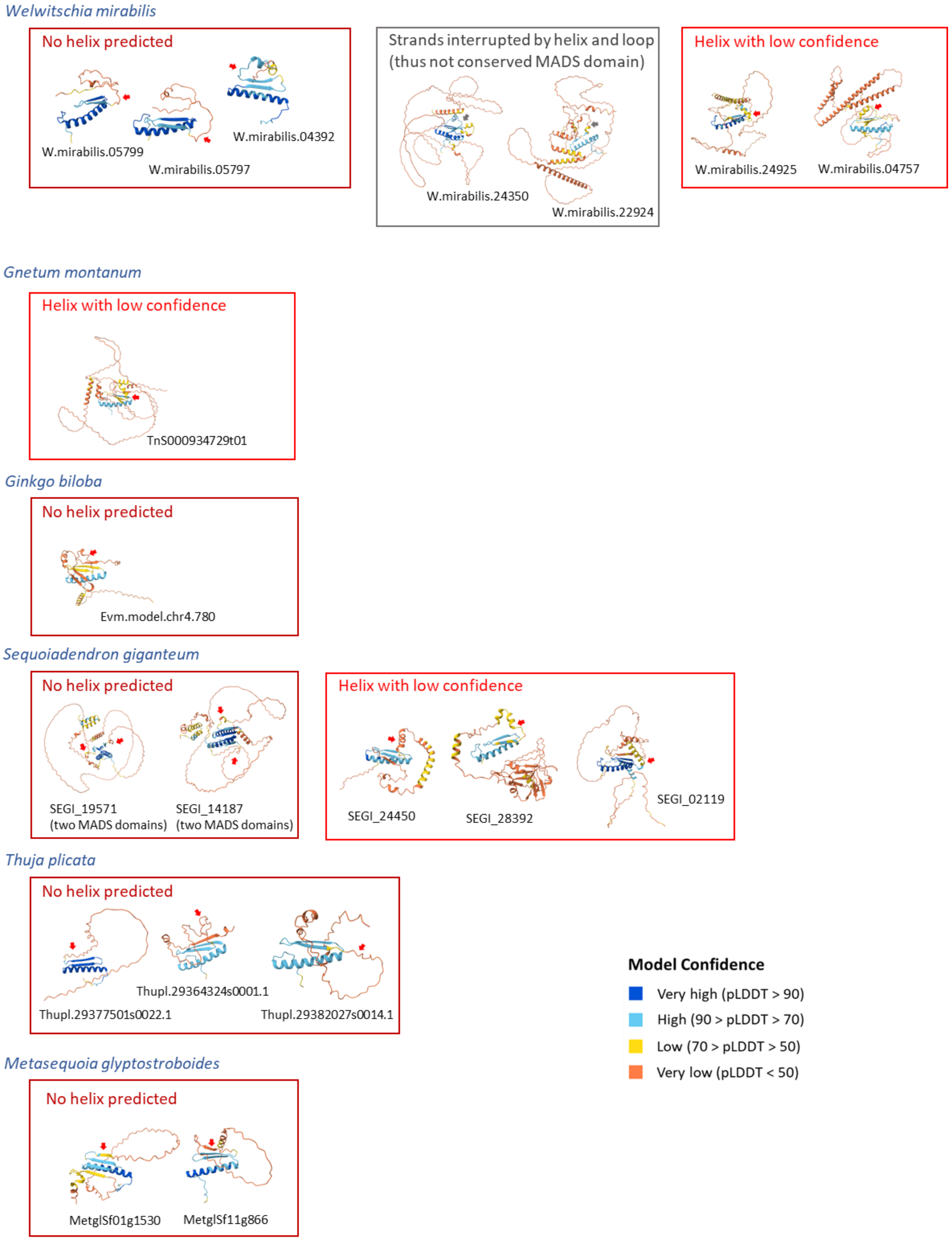

Fig. S6C AlphaFold2-predicted structural models of all 48 "Mα-1" MADS-box proteins (full-length). Red arrows mark the region after the beta strands where a helix is expected. Angiosperm "Mα-1" proteins resemble the MEF2 structure. For the other "Mα-1" proteins, the functional helix is either predicted absent or with low confidence.

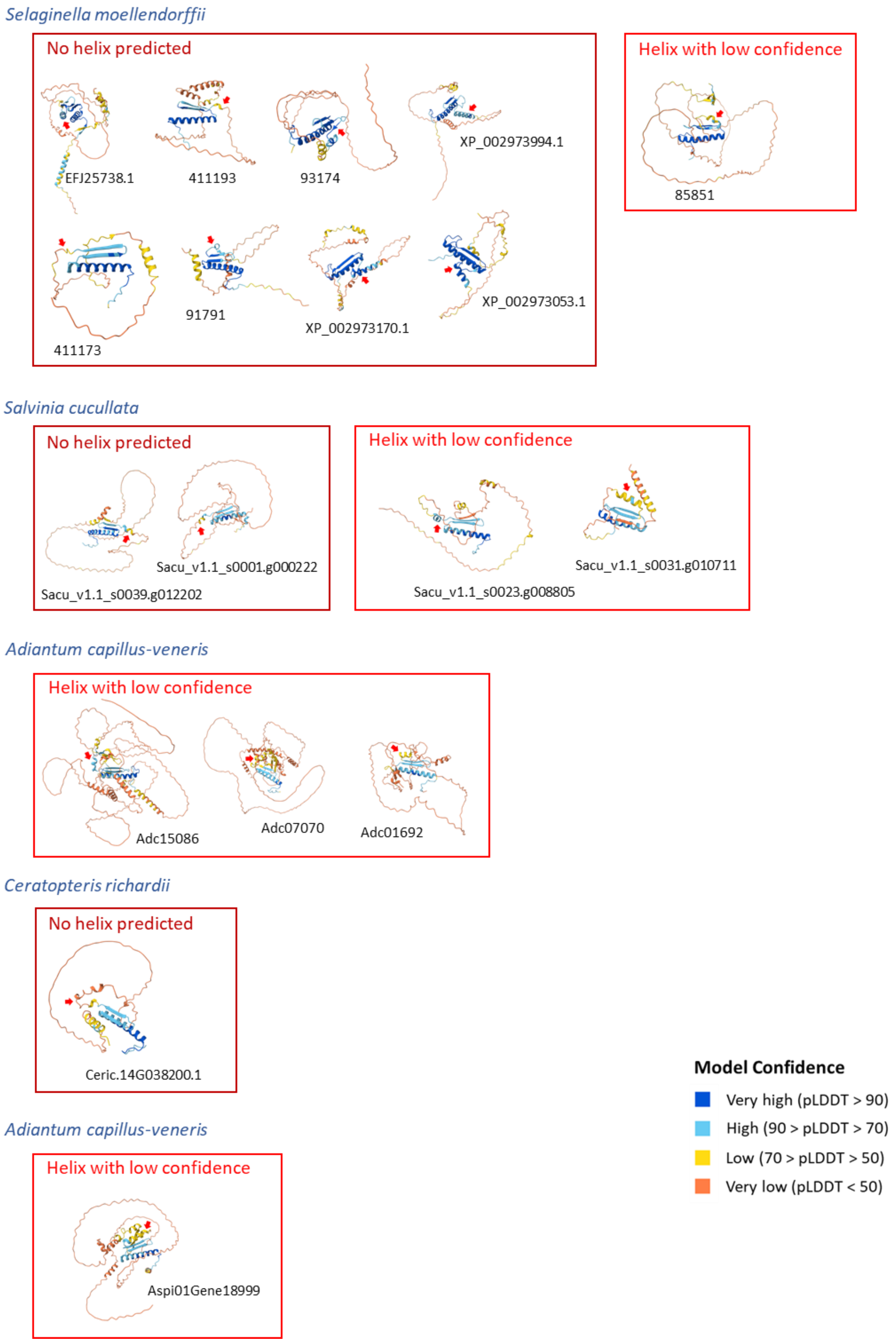

Supplement: evag140_Supplementary_Data [file evag140_supplementary_data.zip › 20260508 GBE revision supplementary figures.pdf]
